# Enhanced phosphate adsorption and desorption characteristics of MgO-modified biochars prepared via direct co-pyrolysis of MgO and raw materials

**DOI:** 10.1186/s40643-023-00670-3

**Published:** 2023-08-10

**Authors:** Panfeng Tu, Guanlin Zhang, Yingyuan Cen, Baoyuan Huang, Juan Li, Yongquan Li, Lifang Deng, Haoran Yuan

**Affiliations:** 1 Institute of Biomass Engineering, South China Agricultural University, Guangzhou, 510642 People’s Republic of China; 2 Zhongkai University of Agriculture and Engineering, Guangzhou, 510225 People’s Republic of China; 3 Guangzhou Institute of Energy Conversion, Chinese Academy of Sciences, Guangzhou, 510640 China

**Keywords:** Co-pyrolysis, MgO-modified biochars, Phosphate adsorption capacity, Phosphate desorption efficiency

## Abstract

Biochar modified by metal ions—particularly Mg—is typically used for the effective recovery of phosphorous. In this study, MgO-modified biochars were synthesized via the direct co-pyrolysis of MgO and raw materials such as rice straw, corn straw, *Camellia oleifera* shells, and branches from garden waste, which were labeled as MRS, MCS, MOT, and MGW, respectively. The resulting phosphate (PO) adsorption capacities and potential adsorption mechanisms were analyzed. The PO adsorption capacities of the biochars were significantly improved after the modification with MgO: MRS (24.71 ± 0.32 mg/g) > MGW (23.55 ± 0.46 mg/g) > MOT (15.23 ± 0.19 mg/g) > MCS (14.12 ± 0.21 mg/g). PO adsorption on the modified biochars was controlled by physical adsorption, precipitation, and surface inner-sphere complexation processes, although no electrostatic attraction was observed. Furthermore, PO adsorbed on modified biochars could be released under acidic, alkaline, and neutral conditions. The desorption efficiency of MRS was modest, indicating its suitability as a slow-release fertilizer.

**Graphical Abstract:**

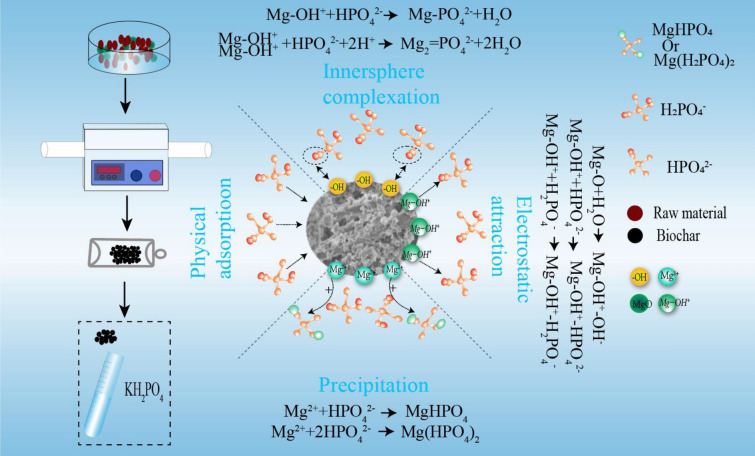

**Supplementary Information:**

The online version contains supplementary material available at 10.1186/s40643-023-00670-3.

## Introduction

Phosphorus is a non-renewable resource widely used as a fertilizer in agriculture. In this context, it is primarily derived from phosphate (PO) ore, which is currently overexploited (Reitzel et al. 2019; Schroder et al. 2011). Additionally, large amounts of PO-containing sewage are being discharged directly into rivers and oceans without treatment, which results in the eutrophication of water bodies and large-scale deterioration of the water quality, harming aquatic life and human health (Peyman et al. 2017). To simultaneously address the problems of water eutrophication and the shortage of PO ore resources, strategies should be developed to recover phosphorous from high-PO-content wastewater for subsequent use in agriculture (Ribeiro et al. 2020).

The standard methods for recovering phosphorous from wastewater include chemical precipitation, biological treatment, adsorption, ion exchange, and electric flocculation (Gao et al. 2019; Yeoman et al. 1988; Zhou et al. 2022). Among these, chemical precipitation has the advantages of simple operation and excellent stability; however, it can cause secondary pollution (Huang et al. 2017). Similarly, biological treatment is low-cost but is characterized by poor stability and reliance on the treatment environment (Bunce et al. 2018). Alternatively, adsorption has attracted attention because it has advantages such as simple operation, low cost, and reusability—particularly when biochar is used as the carrier to adsorb PO from wastewater, which can then be reused in agricultural applications (Bryant et al. 2012). Biochar is a C-rich solid obtained via biomass pyrolysis under anaerobic or hypoxic conditions (Gong et al. 2020; Lehmann et al. 2006). Owing to its abundant functional groups, developed pore structure, and large specific surface area (Liu et al. 2019), biochar is often used as an adsorbent for various pollutants. However, it is a large anion with a negative charge. Thus, it can adsorb positively charged materials, such as heavy-metal ions, but exhibits a poor adsorption ability for anions (Cha et al. 2016; Chun et al. 2021). It has even been reported to it has even been reported to exert a repulsion effect during phosphate and nitrate salt adsorption during PO and nitrate salt adsorption (Pinto et al. 2019). Therefore, physical and chemical modifications are needed to improve the adsorption effect of biochar on anions.

Previous studies have indicated that the PO removal efficiency of biochar can be significantly increased via modification with metal ions. For example, Xu et al. (2019) prepared lignocellulose biochar via LaCl_3_ modification, which exhibited an adsorption capacity of up to 36.06 mg/g for PO adsorption. Furthermore, Wu et al. (2020) modified rice straw-derived biochar with ferrous chloride (Fe(II)) and ferric chloride (Fe(III)), reporting an adsorption capacity of 39.20 mg/g for the Fe(II) biochar. Iron oxide particles were found to enhance the PO adsorption ability while reducing the phosphorous loss. Thus, iron-modified biochar had enhanced availability for PO. Zhang et al. (2022) fabricated a low-cost biochar via co-precipitation modification using seawater as a modifying agent. The as-prepared seawater-modified biochar exhibited a maximum adsorption capacity of 181.07 mg/g for PO owing to the loading of Mg. These findings indicate that the PO adsorption capacity of biochar can be significantly improved via modification with metals—particularly Mg. Notably, Mg is a medium trace element required by plants and has beneficial environmental effects (Hoo et al. 2004). Therefore, the application of Mg-modified biochar in PO adsorption has attracted the attention of researchers.

Modifying biochar by Mg typically involves two steps: (i) soaking in MgCl_2_ and (ii) pyrolysis. Wu et al. (2019) synthesized MgO-modified biochar by combining 50 g of biochar with 1 M MgCl_2_, followed by heating in a muffle furnace at 600 °C, which resulted in a maximum PO adsorption capacity of 18.94 mg/g. Similarly, Fang et al. (2020) prepared Mg/Ca-modified biochars by soaking sugarcane bagasse in MgCl_2_·6H_2_O and CaCl_2_·2H_2_O solutions for 1 h, followed by oven-drying and pyrolysis at 700 °C, achieving an adsorption capacity of up to 129.79 mg/g. These findings indicate that biochar modified via impregnation with MgCl_2_ exhibits a markedly improved PO adsorption capacity; however, this method requires complex technological processes and high costs. Moreover, previous studies have indicated that MgO is the main active functional group during PO adsorption in MgCl_2_-modified biochars (Fang et al. 2022; Li et al. 2022).

The present study addresses the need for a one-step preparation process for MgO-modified biochar for PO adsorption. Four types of agricultural and forestry waste (rice straw, corn straw, *Camellia oleifera* shells, and branches from garden waste) were used as raw materials, which were subjected to co-pyrolysis with MgO to prepare MgO-modified biochars. The PO adsorption capacities and efficiencies of the as-prepared MgO-modified biochars were examined to provide a scientific basis for utilizing agricultural and forestry waste.

## Materials and methods

### Preparation of biochar and Mg-modified biochar

Rice straw, corn straw, *Camellia oleifera* shells, and branches from garden waste were obtained from Guangzhou city in Guangdong province and used as raw materials. They were pre-treated by flushing with tap water to remove dust, followed by washing with distilled water for deionization, and oven-dried for 24 h at 80 °C. The resulting dried raw materials were shredded to 0.5–1.0 cm before use. Next, the pre-treated raw materials were pyrolyzed in a tube furnace at 700 °C for 2 h with a shielding gas of N_2_ and a heating rate of 10 °C/min. After cooling naturally, the samples were washed with deionized water and ethanol three times each. The resulting as-prepared biochars were labeled RS, CS, OT, and GW. MgO-modified biochar was prepared using the same process, except the pre-treated raw material was mixed with MgO at a mass ratio of 3:1 before pyrolysis. The resulting as-prepared biochars were labeled MRS, MCS, MOT, and MGW.

### PO adsorption experiment

#### PO adsorption

Next, the PO adsorption capacities of the as-prepared biochars were determined. Briefly, 0.10 g of as-prepared biochar was mixed with a 100-mg/L KH_2_PO_4_ solution (25 mL, pH = 5.22) in 50-mL centrifuge tubes, followed by mixing in a shaker at 25 °C for 24 h. The samples were then centrifuged, and the resulting supernatants were filtered (0.45 mm) and tested using the ammonium molybdate spectrophotometric method (GB11893-1989). Lastly, the PO adsorption capacity of the samples was calculated according to the difference between the PO contents in the supernatant before and after adsorption using the following equation (Chen et al. 2018):

1 $$ {\text{Q}}_{{\text{e}}}=\left( {{\text{C}}_{{0}} -{\text{C}}_{{\text{e}}} } \right){\text{ V/M}} $$

where Q_e_ represents the PO adsorption capacity (mg/g); C_0_ and C_e_ represent the initial and equilibrium PO concentrations in the aqueous solution, respectively (mg/ L); V represents the volume of PO solution (L); and M represents the mass of the as-prepared biochar sample (g).

#### Kinetic studies

The kinetics of PO adsorption for MRS, MCS, MOT, and MGW were evaluated by diffusing 0.10 g of as-prepared biochar in 50-mL centrifuge tubes containing 25 mL of KH_2_PO_4_ solution (100 mg/L, pH = 5.22). The centrifuge tubes were shaken in a shaker at a constant temperature of 25 °C for 30, 90, 120, 150, 360, 1200, and 1440 min, followed by centrifugation. The resulting supernatants were filtered using a 0.45-μm filter membrane and evaluated using a spectrophotometer (UV-1280; Shimadzu, Japan) at 700 nm.

To quantitatively depict the kinetics of PO adsorption, pseudo-first-order, pseudo-second-order, and intra-particle diffusion models were used (Liu et al. 2022):

2 $$ {\text{pseudo-first-order}{:}\, \text{ln} (\text{q}_\text{e}}-\text{q}_\text{t})=\text{ln}\, \text{q}_\text{e}-\text{k}_{1\text{t}}  $$

3 $$ \text{pseudo-second-order}{:}\,\text{t/q}_{{\text{t}}} = 1/\left( {{\text{k}}_{{2}} {\text{q}}_{{\text{e}}}^{{2}} } \right){\text{ + t/q}}_{{\text{e}}} $$

4 $$ \text{intra-particle diffusion equation}{:}\, \text{q}_{{\text{t}}}  = \text{k}_{{\text{p}}} {\text{t}}^{{1/2}} +{\text{C}} $$

where q_t_ and q_e_ (mg/g) represent the amounts of PO adsorbed at time t and equilibrium, respectively, and k_1_ (min^−1^), k_2_ (g/ (mg min)), and k_p_ (g/(mg min)) are the first-order kinetic rate constant, second-order kinetic rate constant, and apparent diffusion rate constant, respectively. C is a constant (mg/g) that denotes the boundary layer thickness.

#### PO adsorption isotherm analysis

Isotherm analysis was conducted for the kinetic study procedures. Briefly, 0.10 g of MRS, MCS, MOT, and MGW was diffused in 50-mL centrifuge tubes containing 25 mL of KH_2_PO_4_ solution (pH = 5.22) at concentrations of 0, 10, 50, 100, 200, 300, 500, and 1000 mg/L, followed by shaking in a shaker at a constant temperature of 25 °C for 24 h. The Langmuir and Freundlich models were used to quantitatively expound the adsorption isotherms (Li et al. 2016a, b):

5 $$ {\text{Langmuir model: q}}_{{\text{e}}} {\text{ = K}}_{{\text{L}}} {\text{Q}}_{{\text{m}}} {\text{C}}_{{\text{e}}} {/ }\left( {{\text{1 + K}}_{{\text{L}}} {\text{C}}_{{\text{e}}} } \right) $$

6 $$ {\text{Freundlich model: q}}_{{\text{e}}} {\text{ = K}}_{{\text{F}}} {\text{C}}_{{\text{e}}}^{{\text{1/n}}} $$

where C_e_ represents the equilibrium adsorption concentration (mg/L); Q_m_ represents the maximum adsorption capacity (mg/g); K_L_ is the Langmuir constant, which is related to the interaction energies; K_F_ is the Freundlich affinity coefficient, which is related to the adsorption capacity; and *n* is the Freundlich constant, which is related to the adsorption intensity.

### PO desorption experiment

To evaluate the PO desorption performance of the samples, MRS, MCS, MOT, and MGW were soaked in KH_2_PO_4_ solution to produce PO-saturated biochar, and the resulting samples were labeled as MRS–PO, MCS–PO, MOT–PO, and MGW–PO, respectively. Then, 0.10 g of MRS–PO, MCS–PO, MOT–PO, and MGW–PO was suspended in 25 mL of NaOH solution (0.05 mol/L), citric acid solution (2%), or distilled water and oscillated for 24 h before the released PO was measured, as follows:

7 $$ {\text{D}}\,\left( \%\right)=\left( {\text{C*V}} \right){/}\left( {\text{Q*m}} \right) $$

Here, D (%) represents the desorption rate, C represents the PO concentration in the aqueous solution after desorption, V represents the volume of the PO-containing solution (L), Q represents the equilibrium adsorption capacity (mg/g), and m represents the mass of PO-saturated biochar (g).

### Characterization of biochar and Mg-modified biochar

Fourier transform infrared spectroscopy (FTIR) (TENSOR27; Germany) was used to identify the surface functional groups and chemical bonds of the biochar samples before and after PO absorption. Before testing, 0.5 mg of biochar was thoroughly mixed with 100 mg of KBr via grinding in a mortar and then subjected to a pressure of 20 MPa using a tablet press. The spectra were obtained via 60 scans, with wavenumbers ranging from 4000 to 500 cm^−1^. X-photoelectron spectroscopy (XPS) (ESCA Lab 250Xi; USA) was conducted to examine the compositions, chemical states, and electronic states of the elements on the surfaces of the biochar samples. Scanning electron microscopy (SEM) (S-4800; Japan) was conducted to examine the surface morphologies of the as-prepared biochars. The zeta potential of the as-prepared biochar was investigated by dispersing 0.01 g of biochar in 50 mL of deionized water (the solution pH varied from 2.0 to 9.0), followed by 12 h of ultrasonic treatment. The surface charge of the sample was measured using a laser particle size analyzer.

### Statistical method

Origin 2021 was used to calculate the coefficient of determination (R^2^), standard deviation, and other statistical parameters.

## Results and discussion

The PO adsorption capacities of the RS, CS, OT, GW, MRS, MCS, MOT, and MGW biochar samples are presented in Additional file 1: Fig. S1 and Table 1. As unmodified biochar is typically negatively charged and tends to hinder PO adsorption (Yao et al. 2012), far lower adsorption capacities were observed in the four unmodified biochars (RS, CS, OT, and GW). GW exhibited a larger specific surface area, more adsorption sites (Zhang et al. 2020), and a slightly higher PO adsorption capacity (1.87 ± 0.21 mg/g) than the other samples, although the differences were insignificant. In comparison, the PO adsorption capacity of the biochar samples was increased multi-fold after modification with MgO. Among the MgO-modified biochars, MRS exhibited the highest adsorption capacity (24.71 ± 0.32 mg/g), followed by MGW (23.55 ± 0.46 mg/g), MOT (15.23 ± 0.19 mg/g), and MCS (14.12 ± 0.21 mg/g). This result is attributed to the larger specific surface area and the loading of MgO during modification. Although MCS had the largest specific surface area, it also exhibited the lowest PO adsorption capacity. This observation suggests that the relationship between the PO adsorption capacity and specific surface area is not proportional in biochars. Taking into account the adsorption-capacity results, the MgO-modified biochars (MRS, MCS, MOT, and MGW) were selected for further study.

**Table 1:** Special surface areas and adsorption capacities of biochars from rice straw, corn straw, *Camellia oleifera* shells, and branches from garden waste before and after MgO modification

	RS	CS	OT	GW	MRS	MCS	MOT	MGW
Special surface area (m^2^/g)	202.7 ± 5.54	211.22 ± 4.73	301.67 ± 4.72	322.92 ± 7.29	259.33 ± 5.32	373.72 ± 7.19	350.42 ± 7.41	375.74 ± 6.89
Adsorption capacity (mg/g)	0.61 ± 0.05	0.89 ± 0.05	1.35 ± 0.04	1.87 ± 0.09	24.71 ± 0.12	14.12 ± 0.10	15.23 ± 0.11	23.55 ± 0.12

### Characteristics of as-prepared biochars

#### FTIR analysis

**Fig. 1:**
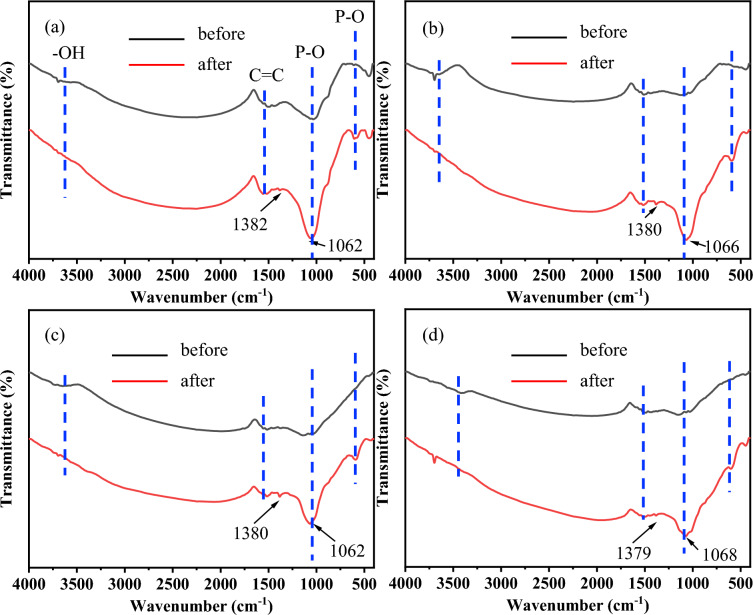
FTIR spectra of **a** MRS, **b** MCS, **c** MOT, and **d** MGW before and after PO adsorption

FTIR is commonly used to characterize the functional groups of samples (Bekiaris et al. 2016). In this study, FTIR was used to evaluate the PO adsorption and the transformation process in biochar. As shown in Fig. 1, clear absorption bands appeared at approximately 1064 ± 5 cm^−1^ and 591 ± 3 cm^−1^ for MRS, MCS, MOT, and MGW after PO desorption, which were assigned to the P–O asymmetric stretching vibration of the PO_4_^3−^ group and the bending vibration of O–P–O, respectively (Li et al. 2020; Wang, et al. 2018). Additionally, weak peaks appeared at approximately 591 ± 3 cm^−1^ before PO adsorption, and the peaks at approximately 1064 ± 5 cm^−1^ for MRS, MCS, MOT, and MGW are attributed to the stretching and bending vibrations of Mg–O and Mg–OH (Tongamp et al. 2007). The peak intensity at 1064 ± 5 cm^−1^ for MRS, MCS, MOT, and MGW increased after PO desorption, indicating that these biochars successfully adsorbed additional PO ions. The peaks at approximately 1553 ± 3 cm^−1^ may correspond to C=C vibrations (Liao et al. 2018). Additionally, the weak peaks observed at approximately 3372–3525 cm^−1^ and 1381 ± 8 cm^−1^, which are attributed to –OH bending vibrations, nearly disappeared after PO adsorption, indicating the interaction between PO and the –OH group (Liao et al. 2018).

#### XPS analysis

XPS—an effective method for analyzing the types and forms of elements present on the surfaces of materials—was used to evaluate the different biochar samples (Yang et al. 2009). The obtained XPS spectra of the unmodified biochars (RS, CS, OT, and GW) and the MgO-modified biochars (MRS, MCS, MOT, and MGW) before and after the PO adsorption are presented in Additional file 1: Fig. S2. As shown in Additional file 1: Fig. S2 and Table S1, all the biochars were found to be rich in C and O. Furthermore, higher levels of Mg were detected in MRS, MCS, MOT, and MGW compared with the unmodified biochars (RS, CS, OT, and GW), indicating the successful loading of Mg in biochars via the co-pyrolysis of MgO with biomass. Notably, MRS exhibited the highest Mg loading capacity (11.90 at.%), which explains why it had the highest adsorption capacity. Moreover, the peak intensities of PO (136.36 ± 0.19 eV) were increased after PO adsorption, which confirmed the successful adsorption of PO in MRS, MCS, MOT, and MGW and was consistent with previous results.

**Fig. 2:**
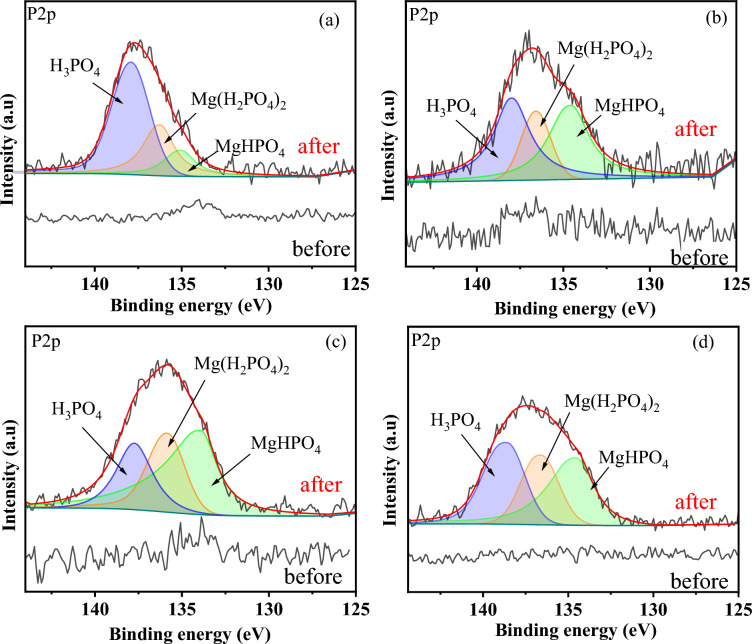
High-resolution XPS spectra of P2p for **a** MRS, **b** MCS, **c** MOT, and **d** MGW before and after PO adsorption

The XPS spectra of P2p were evaluated in MRS, MCS, MOT, and MGW before and after PO adsorption. As shown in Fig. 2, although the P2p peaks for MRS, MCS, MOT, and MGW before PO adsorption were negligible, clear peaks appeared after PO adsorption (range 125 to 145 eV) at binding energies of 134.96 ± 0.23, 136.36 ± 0.19, and 138.08 ± 0.21 eV, which were assigned to PO_4_^3−^, HPO_4_^2−^, and PO analogs, respectively (Yao et al. 2013; Zhu, et al. 2020). These results indicate that PO adsorption is due to the reaction of PO anions and MgO, as well as surface deposition (Yao et al. 2013). To confirm these findings, the Mg1s spectra for MRS, MCS, MOT, and MGW after PO desorption were evaluated. As shown in Additional file 1: Fig. S2, peaks were observed at binding energies of 1308.38 ± 0.30, 1306.00 ± 0.34, and 304.28 ± 0.32 eV, which were assigned to MgHPO_4_, Mg(H_2_PO_4_)_2_, and MgO (Zhu et al. 2020), respectively, confirming the above results.

#### SEM

**Fig. 3:**
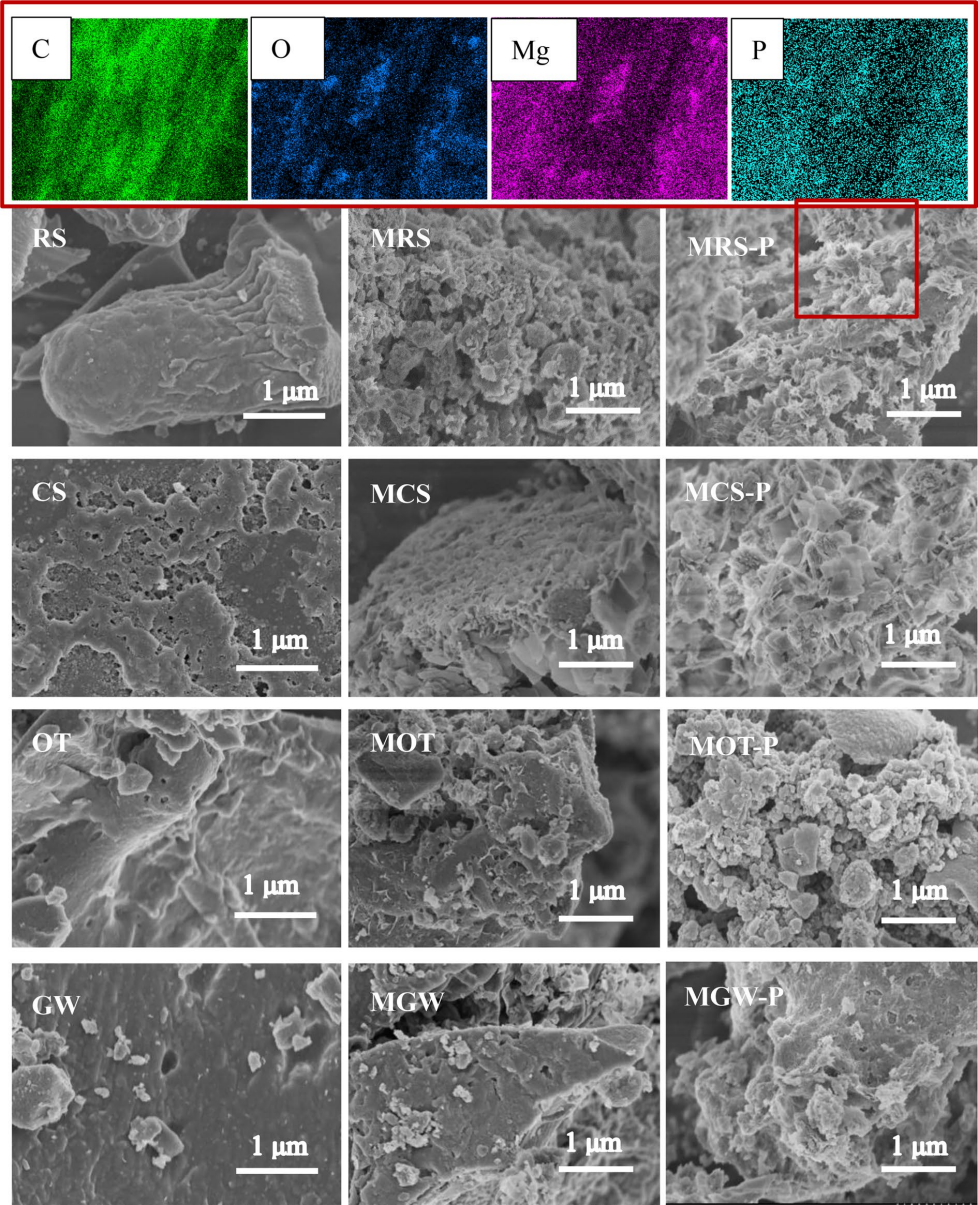
SEM images of unmodified biochars (RS, CS, OT, and GW), modified biochars (MRS, MCS, MOT, and MGW), and PO-loaded biochars (MRS–PO, MCS–PO, MOT–PO, and MGW–PO) and EDX mapping of MRS–PO

SEM images of the unmodified biochars (RS, CS, OT, and GW), modified biochars (MRS, MCS, MOT, and MGW), and PO-loaded biochars (MRS–PO, MCS–PO, MOT–PO, and MGW–PO) are presented in Fig. 3. As shown, the unmodified biochars had smooth surfaces, indicating small specific surface areas (Table 1). The modified biochars exhibited rougher and more porous morphologies, which is attributed to the pyrolysis and the deposition of MgO nanoparticles during co-pyrolysis (Zhu et al. 2020). Moreover, clusters of flakes were observed on the PO-loaded biochars (MRS–PO, MCS–PO, MOT–PO, and MGW–PO). Energy-dispersive X-ray spectroscopy (EDX) mapping of MRS–PO confirmed the homogenous distribution of C, O, Mg, and P. Together with the XPS results, these findings confirm the formation of MgHPO_4_ and Mg(H_2_PO4)_2_ on the surfaces of the MgO-modified biochars.

#### Point of zero charge (pH_pzc_)

Previous studies have indicated that both the PO species and the adsorption reactions vary with respect to the pH (Li et al. 2016a, b; Wu et al. 2020). In this context, negatively charged H_2_PO_4_^−^ was predominant at pH values of 2.10–7.20, whereas HPO_4_^2−^ was the dominant species at pH values of 7.20–12.30. PO_4_^−^ may exist in large quantities at pH values of 10.67–12. Moreover, inner-sphere PO complex formation and ligand exchange are more likely to occur in H_2_PO_4_^−^ compared with HPO_4_^2−^. This is attributed to the far higher adsorption-free energy of HPO_4_^2−^ (Li et al. 2019; Lin et al. 2019). Hence, the pH of solutions significantly affects the PO adsorption. According to Liu et al. (2016), when pH < pH_pzc_, Mg oxides on the surfaces of the biochars become protonated and synthesise MgOH^+^, increasing the pH of the solution. Biochars with MgOH^+^ on their surfaces are inclined to bond with PO ions via electrostatic attraction. As the pH increases, the protonation effect of Mg oxides gradually becomes weaker, and the biochar surface becomes negatively charged, leading to an electrostatic interaction that is repulsive toward PO anions and a reduced PO adsorption capacity, which results in chemical precipitation and complexation reactions (Li et al. 2019; Li, et al. 2016a, b). The zeta potentials of MRS, MCS, MOT, and MGW at different pH values are presented in Additional file 1: Fig. S3. The corresponding points of zero charge (pH_pzc_) were 4.19, 2.01, 3.07, and 2.23, respectively. MRS exhibited the highest pHpzc among the samples, confirming its highest PO adsorption capacity. The pH of the KH_2_PO_4_ solution (pH = 5.22) was higher than the pH_pzc_ values of MRS, MCS, MOT, and MGW, indicating poor (or a lack of) electrostatic interactions in the MgO-modified biochars. The previously observed PO desorption may have been due to chemical precipitation and complexation reactions.

### PO adsorption mechanism

#### Kinetics of PO adsorption

**Fig. 4:**
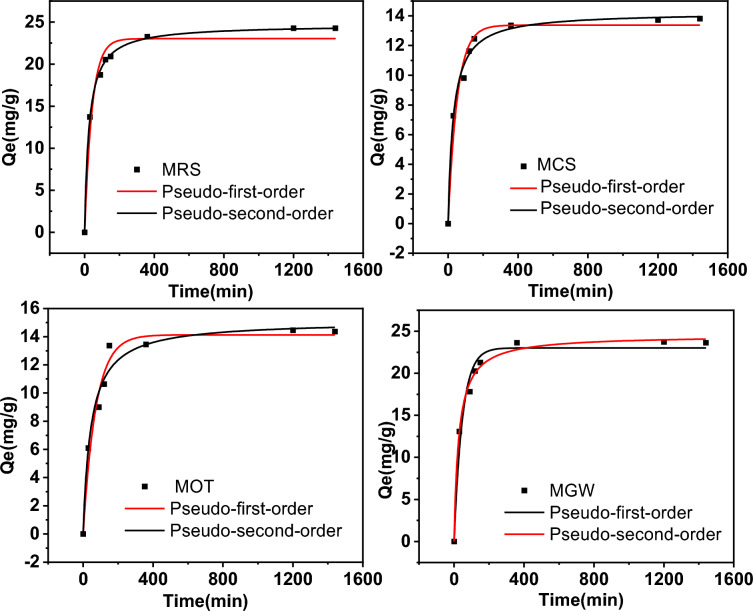
PO adsorption kinetics for MRS, MCS, MOT, and MGW. Symbols indicate the experimental data, and the lines indicate the results of the simulations based on the pseudo-first-order and pseudo-second-order models

Adsorption kinetics describe the retention rate of a solute under given conditions and reflect the retention time of an adsorption unit to achieve a required solute concentration, which indicates the adsorption speed of a material. The PO adsorption kinetics of the unmodified biochar (RS) and modified biochars (MRS, MCS, MOT, and MGW) at an initial concentration of 100 mg/L were analyzed, and the results are presented in Fig. 4 and Additional file 1: Fig. S5. MRS, MCS, MOT, and MGW exhibited a two-stage absorption process. After PO adsorption in the first stage (0–150 min), the adsorption rate increased significantly and reached saturation in the second stage (360–1440 min). The adsorption rate of the modified biochars in this paper was far more rapid than that of MgCl_2_-modified biochars derived from ground coffee waste and corn stalks (Shin et al. 2020; Zhu et al. 2020). The rapid adsorption observed in the first stage is attributed to electrostatic forces and the rapid precipitation reaction on the surfaces of the biochar samples. In contrast, the slow adsorption in the second stage indicated the occurrence of the physical adsorption and intra-particle diffusion of the PO dissolved into the biochar (Fang et al. 2020; Yang et al. 2021a, b). However, RS was found to absorb little PO and only exhibited a slow one-stage absorption process, which is attributed to the physical adsorption (Additional file 1: Fig. S4).

Next, the absorption data for RS, MRS, MCS, MOT, and MGW were further analyzed via fitting with pseudo-first-order and pseudo-second-order models. These models agreed with the experimental data. They exhibited significant correlation coefficients, indicating that both physical and chemical adsorption occurred (Wu et al. 2020). Moreover, the correlation coefficients of the pseudo-second-order model for MRS (R^2^ = 0.998), MCS (*R*^2^ = 0.990), MOT (R^2^ = 0.971), and MGW (R^2^ = 0.994) exceeded those of the pseudo-first-order model (Table 2). The pseudo-second-order model may be better suited to describe PO adsorption by MRS, MCS, MOT, and MGW, as the Q_e_ values were closer to the experimental values. Thus, PO adsorption mainly occurred via chemisorption in MRS, MCS, MOT, and MGW (Buates and Imai 2020). MRS exhibited the highest adsorption capacity and smallest specific surface area, and MCS exhibited the lowest adsorption capacity and largest specific surface area, confirming the dominance of chemisorption in MRS, MCS, MOT, and MGW. However, RS appeared to fit the pseudo-first-order model more closely, indicating the occurrence of intra-particle diffusion and physical processes (Additional file 1: Table S2) (Jung et al. 2015).

**Table 2:** Kinetic parameters of PO adsorption in MRS, MCS, MOT, and MGW based on pseudo-first-order and pseudo-second-order models

Materials	Pseudo first-order			Pseudo second-order		
	q_e_ (mg/g)	K_1_/min^−1^	R^2^	q_e_ (mg/g)	K_2_/min^−1^	R^2^
MRS	23.031	0.024	0.967	24.686	0.040	0.998
MCS	13.375	0.019	0.970	14.241	0.033	0.990
MOT	14.192	0.013	0.967	15.110	0.022	0.971
MGW	23.019	0.021	0.971	24.052	0.037	0.994

#### Isotherm of PO adsorption

**Fig. 5:**
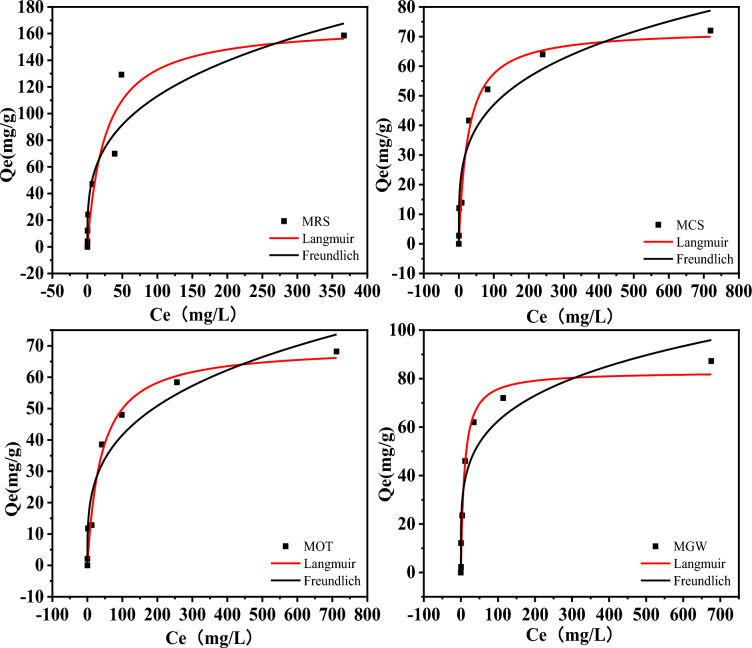
PO adsorption isotherms for MRS, MCS, MOT, and MGW. Symbols indicate the experimental data, and the lines indicate the results of the simulations based on the Freundlich and Langmuir models

Adsorption isotherms reflect the interactions between adsorbates and adsorbents and are thus crucial for characterizing the capacity of adsorbents from which the corresponding adsorption systems can be derived. Adsorption isotherm analysis was performed to gain insights into the PO adsorption properties of MRS, MCS, MOT, and MGW. The data corresponding to this analysis are presented in Fig. 5. The intensity of PO adsorption increased with an increasing concentration of PO in the solution. Furthermore, although the adsorption isotherms of PO were nonlinear, they exhibited a concave downward shape, and the PO adsorption capacities of MRS, MCS, MOT, and MGW increased with the equilibrium concentration.

To examine the differences in PO adsorption capacity between the different biochar samples, two-parameter equations, i.e., the Langmuir and Freundlich models, corresponding to the homogenous and heterogeneous sorbent surfaces, respectively, were used to fit the experimental data (Ifthikar et al. 2017). Figure 5 and Table 3 show that both models adequately reproduced the adsorption data, indicating a strong interaction between PO and the biochar samples. The Langmuir model exhibited a correlation coefficient (*R*^*2*^) of 0.91–0.99, which exceeded that of the Freundlich model (0.86–0.94), indicating that the Langmuir model was a better fit for the experimental data. The model assumed monolayer PO adsorption in MRS, MCS, MOT, and MGW, with MgO reacting with PO on the homogeneous surfaces of the four modified biochars. MRS exhibited the highest theoretical PO adsorption capacity (167.29 mg/g), which was approximately 2.02, 2.31, and 2.39 times higher than those of MGW (82.90 mg/g), MCS (72.49 mg/g), and MOT (69.86 mg/g), respectively, and significantly higher than those of most previously reported biochars (Additional file 1: Table S3). These results indicate that MgO co-pyrolysis can efficiently provide a high PO adsorption capacity during biochar modification. Additionally, the correlation coefficients in the Langmuir model were < 0.95, whereas the n values were > 3. This suggests that all four modified biochars (MRS, MCS, MOT, and MGW) had high capacities for PO adsorption, which was dominated by monolayer adsorption (Zhang et al. 2015).

**Table 3:** Isotherm parameters of PO adsorption in MRS, MCS, MOT, and MGW based on Freundlich and Langmuir models

	Langmuir			Freundlich		
	Q_m_ (mg/g)	K_L_ (L/mg)	R^2^	n	K_f_	R^2^
MRS	167.290	0.039	0.911	3.296	27.96154	0.905
MCS	72.485	0.038	0.972	3.816	14.05847	0.930
MOT	69.860	0.025	0.975	3.430	10.85208	0.940
MGW	82.906	0.105	0.981	4.457	22.23913	0.900

#### Adsorption mechanism analysis

Previous studies have indicated that the processes of physical adsorption, precipitation, electrostatic attraction, and inner-sphere complexation contribute to PO adsorption in Mg-modified biochars (Li et al. 2016a, b; Wu et al. 2019; Yao et al. 2013). According to the results of the PO adsorption capacity and adsorption kinetics analysis, RS with a larger specific surface area had a higher PO adsorption capacity, indicating that physical adsorption may occur in biochars with large specific surface areas and rich pore structures. The XPS results indicated the existence of Mg(H_2_PO_4_)_2_ and MgHPO_4_ on the surfaces of the biochar samples after PO adsorption, which was attributed to the precipitation of Mg oxyhydroxides upon their reaction with HPO_4_^2−^ and H_2_PO_4_^−^. Furthermore, the clusters of flakes observed on the surfaces of the PO-loaded biochars confirmed the precipitation of Mg–PO. In addition, the FTIR results highlighted the asymmetric vibration of P–O bonds at 1064 ± 5 cm^−1^, indicating the interaction between the Mg–OH group and PO ions and the occurrence of surface inner-sphere complexation processes. However, as the surfaces of the biochar samples were negatively charged when pH > pH_PZC_ (KH_2_PO_4_ solution, pH = 5.52), electrostatic attraction was unlikely.

**Fig. 6:**
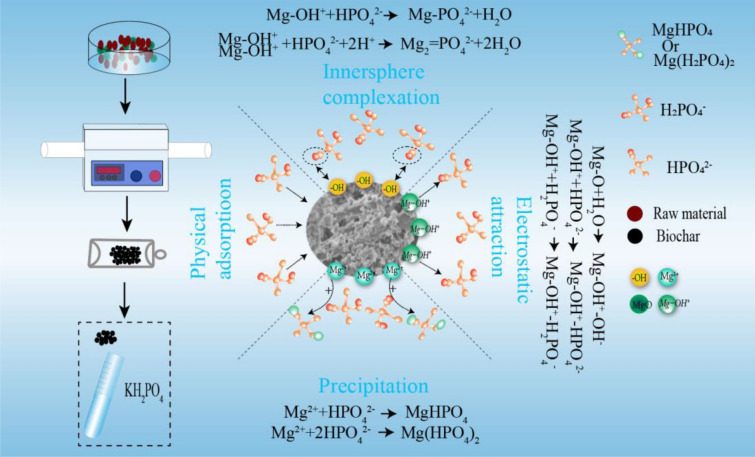
Mechanisms of PO adsorption in MRS

These results suggest that PO adsorption on the MgO-modified biochars (prepared via the co-pyrolysis of MgO with biomass) was controlled by a combination of physical adsorption, precipitation, and surface inner-sphere complexation processes. Additionally, no electrostatic attraction was observed (Fig. 6).

### PO desorption efficiency

**Fig. 7:**
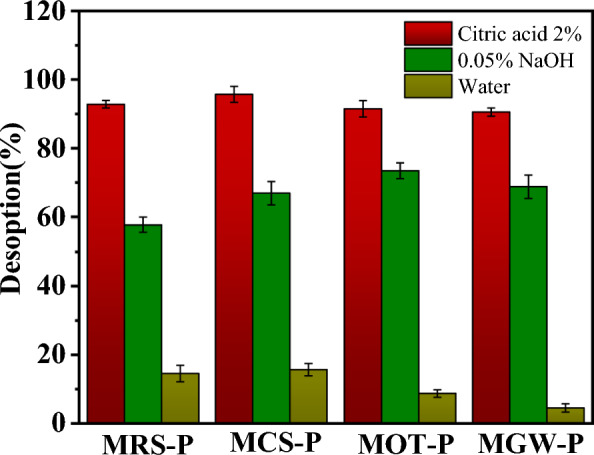
PO desorption efficiencies of MRS–PO, MCS–PO, MOT–PO, and MGW–PO in 2% citric acid, 0.05% NaOH, and water

PO desorbability is crucial for the practical application of MgO-modified biochars as PO-loading carriers and the subsequent use of recovered PO as fertilizer in agriculture. In this study, to determine the PO desorbability of the MgO-modified biochars, the PO desorption efficiencies of MRS–PO, MCS–PO, MOT–PO, and MGW–PO were evaluated in 2% citric acid, 0.05% NaOH, and water (Fig. 7). Phosphate was found to be effectively released by MRS, MCS, MOT, and MGW in all the solutions. Regarding the high solubility of Mg(H_2_PO_4_)_2_ and MgHPO_4_ under acidic conditions (Shin et al. 2020; Yang et al. 2021a, b) and the dissolvability of the magnesium PO compounds in citric acid (Nardis et al. 2022), a PO desorption efficiency of up to 90.53–95.71% was observed in 2% citric acid, which was higher than those in 0.05% NaOH (57.83–73.44%) and water (4.52–14.54%). These results indicate that acidic and alkaline conditions were adequate for PO desorption, and that the PO desorption efficiencies of MRS, MCS, MOT, and MGW differed among the solutions tested, suggesting that the interplay between PO and the biochars differed. Moreover, MCS exhibited the highest PO desorption efficiency at 2% citric acid. In contrast, most PO was released by MOT in 0.05% NaOH, and MRS exhibited the highest desorption in water. In the context of a slow-release fertilizer, if PO is released too slowly or too quickly, its value with regard to agricultural usage is reduced. Thus, owing to its PO adsorption capacity, MRS is deemed the most suitable material for this application.

## Conclusion

Biomass in the form of rice straw, corn straw, *Camellia oleifera* shells, and branches from garden waste was used as feedstock to prepare MgO-modified biochars (MRS, MCS, MOT, and MGW) via one-step co-pyrolysis. Because of the large corresponding specific surface areas and MgO loads, the resulting MgO-modified biochars exhibited significantly higher PO adsorption capacities than unmodified biochars: MRS (24.71 ± 0.32 mg/g) > MGW(23.55 ± 0.46 mg/g) > MOT (15.23 ± 0.19 mg/g) > MCS (14.12 ± 0.21 mg/g). The phosphate adsorption in these MgO-modified biochars followed pseudo-second-order kinetics, suggesting that PO adsorption mainly occurred via chemisorption. Furthermore, the isotherm data fit well with the Langmuir and Freundlich models, indicating an effective interaction between PO and the biochar samples evaluated. XPS results indicated the existence of Mg(H_2_PO_4_)_2_ and MgHPO_4_ on the surfaces of the biochar samples after PO adsorption, which was attributed to the precipitation of Mg oxyhydroxides that reacted with HPO_4_^2−^ and H_2_PO_4_^−^. Moreover, clusters of flakes observed on the surfaces of the PO-loaded biochars indicated the precipitation of Mg–PO. FTIR spectra indicated that the interactions between the Mg–OH group and PO ions occurred via surface inner-sphere complexation processes. Additionally, PO adsorbed by the MgO-modified biochars could be released under acidic, alkaline, or neutral conditions. As the desorption efficiency of MRS was modest, MgO-modified biochars obtained from rice straw are likely to be suitable for use as a slow-release fertilizer.

### Supplementary Information

**Additional file 1: Table S1.** Percentage of the surface elements calculated based on the XPS results (at. %) **Table S2.** Parameters of adsorption kinetic models. **Table S3.** Comparison of adsorption properties of different adsorbents for phosphate adsorption. **Figure S1.** P adsorption capacity of the as-prepared biochars. **Figure S2.** XPS spectras of unmodified biochars (RS, CS, OT and GW), MgO modified biochars (MRS, MCS, MOT and MGW) before and after the P adsorption. **Figure S3.** High-resolution XPS spectra of Mg1s after P desorption: (a) MRS, (b) MCS, (c) MOT, and (d) MGW. **Figure S4.** Zeta potentials of MRS, MCS, MOT and MGW at different pH. **Figure S5.** P adsorption kinetics and models for unmodified biochar (RS) and MgO modified biochar (MRS).

## Data Availability

All data supporting this article’s conclusion are available.
