# Supplementary material for: Enhanced phosphate adsorption and desorption characteristics of MgO-modified biochars prepared via direct co-pyrolysis of MgO and raw materials
Source: Bioresour Bioprocess. 2023 Aug 10;10(1):49. doi: 10.1186/s40643-023-00670-3 (PMC10991339; doi:10.1186/s40643-023-00670-3)
Supplement: Supplementary file 1 — Additional file 1: Table S1. Percentage of the surface elements calculated based on the XPS results (at. %) Table S2. Parameters of adsorption kinetic models. Table S3. Comparison of adsorption properties of different adsorbents for phosphate adsorption. Figure S1. P adsorption capacity of the as-prepared biochars. Figure S2. XPS spectras of unmodified biochars (RS, CS, OT and GW), MgO modified biochars (MRS, MCS, MOT and MGW) before and after the P adsorption. Figure S3. High-resolution XPS spectra of Mg1s after P desorption: (a) MRS, (b) MCS, (c) MOT, and (d) MGW. Figure S4. Zeta potentials of MRS, MCS, MOT and MGW at different pH. Figure S5. P adsorption kinetics and models for unmodified biochar (RS) and MgO modified biochar (MRS). [file 40643_2023_670_MOESM1_ESM.docx]

**Additional file**

Enhanced phosphate adsorption and desorption characteristics by MgO-modified biochars prepared via the direct co-pyrolysis of MgO and raw materials

Panfeng Tu ^b^, Guanlin Zhang ^b, c^, Yingyuan Cen ^b^, Baoyuan Huang ^a^, Juan Li ^b^, Yongquan Li ^b^, Lifang Deng ^a*^, Haoran Yuan ^a,c^

^a^ Institute of Biomass Engineering, South China Agricultural University, Guangzhou 510642, P.R. China.

^b^ Zhongkai University of Agriculture and Engineering, Guangzhou 510225, P.R. China.

^c^ Guangzhou Institute of Energy Conversion, Chinese Academy of Sciences, Guangzhou 510640, China.

**^*^ Corresponding author:** Lifang Deng, Email: [nannandeng@163.com](mailto:nannandeng@163.com)

Table S1 Percentage of the surface elements calculated based on the XPS results (at. %)

| Element (at. %) | MRS | MRS-P | MCS | MCS-P | MOT | MOT-P | MGW | MGW-P |
| --- | --- | --- | --- | --- | --- | --- | --- | --- |
| C | 55.88 | 32.81 | 53.20 | 47.83 | 62.62 | 60.27 | 62.76 | 60.77 |
| O | 31.84 | 50.33 | 36.51 | 37.80 | 27.44 | 30.46 | 26.00 | 31.8 |
| P | 0.38 | 3.73 | 0.47 | 0.91 | 0.19 | 1.04 | 0.29 | 2.20 |
| Mg | 11.90 | 13.13 | 9.82 | 13.46 | 9.75 | 8.23 | 10.95 | 5.23 |

Table S2 Parameters of adsorption kinetic models

|  | pseudo first-order | | | pseudo second-order | | |
| --- | --- | --- | --- | --- | --- | --- |
|  | Qe/（mg/g） | K_1_/h^-1^ | R^2^ | Qe/(mg/g) | K_2_/h^-2^ | R^2^ |
| MRS | 23.031 | 0.024 | 0.967 | 24.686 | 0.040 | 0.998 |
| RS | 0.611 | 0.011 | 0.988 | 0.569 | 0.020 | 0.946 |

Table S3 Comparison of adsorption properties of different adsorbents for phosphate adsorption

| Material | Modification method | Adsorbate | Maximum P adsorption capacity（mg/g） | | References |
| --- | --- | --- | --- | --- | --- |
| Saw dust | Co-pyrolysis of limestone and slag | phosphate | 15 | (Yang et al., 2021) | |
| Crofton weed | - | phosphate | 2.32 | (Cheng et al., 2021) | |
| Lignocellulose | LaCl_３_ | phosphate | 36.06 | (Xu et al., 2019) | |
| Carrot pomace | MgCl_2_ Impregnation | phosphate | 138 | (Pinto et al., 2019) | |
| Corn straw | MgCl_2_ Impregnation | phosphate | 60.95 | (Zhu et al., 2020) | |
| Ground coffee waste | MgCl_2_ Impregnation | phosphate | 63.51 | (Shin et al., 2020) | |
| Corn cob | MgCl_2_ Impregnation | phosphate | 8.55 | (Jena et al., 2021). | |
| Bamboo | MgCl_2_ Impregnation | phosphate | 121.8 | (Zheng et al., 2020) | |
| Sewage sludge | CaCl_2_ Impregnation | phosphate | 168.7 | (Saadat et al., 2018) | |
| Fir wood waste | Seawater Co-precipitation | phosphate | 181.07 | (Zhang et al., 2022) | |
| MRS | Co-pyrolysis of MgO and rice straw | phosphate | 167.29 | This study | |

**References**

CHENG N, WANG B, FENG Q W, et al. 2021. Co-adsorption performance and mechanism of nitrogen and phosphorus onto eupatorium adenophorum biochar in water. Bioresource Technology [J], 340.

JENA J, DAS T, SARKAR U 2021. Explicating proficiency of waste biomass-derived biochar for reclaiming phosphate from source-separated urine and its application as a phosphate biofertilizer. Journal of Environmental Chemical Engineering [J], 9.

PINTO M D E, DA SILVA D D, GOMES A L A, et al. 2019. Biochar from carrot residues chemically modified with magnesium for removing phosphorus from aqueous solution. Journal of Cleaner Production [J], 222: 36-46.

SAADAT S, RAEI E, TALEBBEYDOKHTI N 2018. Enhanced removal of phosphate from aqueous solutions using a modified sludge derived biochar: Comparative study of various modifying cations and RSM based optimization of pyrolysis parameters. Journal of Environmental Management [J], 225: 75-83.

SHIN H, TIWARI D, KIM D J 2020. Phosphate adsorption/desorption kinetics and P bioavailability of Mg-biochar from ground coffee waste. Journal of Water Process Engineering [J], 37.

XU Q Y, CHEN Z B, WU Z S, et al. 2019. Novel lanthanum doped biochars derived from lignocellulosic wastes for efficient phosphate removal and regeneration. Bioresource Technology [J], 289.

YANG S M, KATUWAL S, ZHENG W, et al. 2021. Capture and recover dissolved phosphorous from aqueous solutions by a designer biochar: Mechanism and performance insights. Chemosphere [J], 274.

ZHANG M D, HE M Z, CHEN Q P, et al. 2022. Feasible synthesis of a novel and low-cost seawater-modified biochar and its potential application in phosphate removal/recovery from wastewater. Science of the Total Environment [J], 824.

ZHENG Y L, ZIMMERMAN A R, GAO B 2020. Comparative investigation of characteristics and phosphate removal by engineered biochars with different loadings of magnesium, aluminum, or iron. Science of the Total Environment [J], 747.

ZHU D C, CHEN Y Q, YANG H P, et al. 2020. Synthesis and characterization of magnesium oxide nanoparticle-containing biochar composites for efficient phosphorus removal from aqueous solution. Chemosphere [J], 247.

Figure S1 P adsorption capacity of the as-prepared biochars

Figure S2 XPS spectras of unmodified biochars (RS, CS, OT and GW), MgO modified biochars (MRS, MCS, MOT and MGW) before and after the P adsorption

**Figure** S**3** High-resolution XPS spectra of Mg1s after P desorption: (a) MRS, (b) MCS, (c) MOT, and (d) MGW.

Figure S4 Zeta potentials of MRS, MCS, MOT and MGW at different pH

Figure S5 P adsorption kinetics and models for unmodified biochar (RS) and MgO modified biochar (MRS)
